# Defective Morphogenesis and Functional Maturation in Fetal Islet-Like Cell Clusters From OLETF Rat, A Model of NIDDM

**DOI:** 10.1155/EDR.2000.289

**Published:** 2000

**Authors:** Min Zhu, Akira Mizuno, Yoshihiko Noma, Takashi Murakami, Masamichi Kuwajima, Kenji Shima, Michael S. Lan

**Affiliations:** 1 Research Institute for Children Children's Hospital Departments of Pediatrics and Genetics Louisiana State University Health Sciences Center New Orleans LA 70112 USA; 2 Department of Laboratory Medicine Tokushima University Medical School Tokushima City Japan; 3 Research Institute for Children 520 Elmwood Park Blvd. Suite 160 Harahan LA 70123 USA

## Abstract

A failure in the compensate proliferation of pancreatic *β*-cells, as the primary pathogenic event, has been reported in OLETF rat, a model of NIDDM. The aim of the present study is to define whether the *β*-cell defect is attributed to the fetal stage islet development, if so, whether the defect involves down regulation of PDX-1 protein expression. Morphological changes, *β*-cell function, and the expression of PDX-1 protein were examined in the cultured fetal islet-like cell clusters (ICCs) from OLETF rats along with their diabetes-resistant control counterpart LETO rats in the presence of 5.5 or 11.1mM glucose for 48, 72, 96, and 120-hr, respectively. We have observed four abnormalities in the ICCs of OLETF rats. First, a defective morphogenesis was noted during the 72 to 120-hr ICC culture, a period characterized by a dramatic increase in both *β*-cell and non-*β*-cell (*α*,σ, and PP) populations in control rats. This defective morphogenesis was demonstrated by a growth retardation of epithelial stratification and poor development of both *β*-cell and non-*β*-cell masses along with a parallel decline in relevant islet hormone contents. Second, a functional defect was characterized by failure to response to glucose during the 96 to 120- hr-cultured ICCs. Third, the ultrastructural analysis revealed a significant reduction in the number of secretory granules. Four, Western blot analysis showed a significant decrease of PDX-1 protein expression in the OLETF ICCs cultured in 11.1mM glucose for 48 to 72-hr and in 5.5mM glucose for 120-hr. Therefore, we concluded that during the fetal stage of islet development, OLETF rats exhibit both morphological and functional defects.


Int. Jnl. Experimental Diab. Res., Vol. 1, pp. 289-298
Reprints available directly from the publisher
Photocopying permitted by license only

© 2001 OPA (Overseas Publishers Association) N.V.
Published by license under
the Harwood Academic Publishers imprint,
part of The Gordon and Breach Publishing Group.
Printed in Malaysia.

### Defective Morphogenesis and Functional Maturation in Fetal Islet-like Cell Clusters from OLETF Rat, a Model of NIDDM

MIN ZHU[a], AKIRA MIZUNO[b], YOSHIHIKO NOMA[b], TAKASHI MURAKAMI[b], MASAMICHI KUWAJIMA[b], KENJI SHIMA[b] and MICHAEL S. LAN[a,*]

[a]*Research Institute for Children, Children's Hospital, Departments of Pediatrics and Genetics, Louisiana State University Health Sciences Center, New Orleans, LA 70112, USA;*
[b]*Department of Laboratory Medicine, Tokushima University Medical School, Tokushima City, Japan*

*(Received 29 June 2000; In final form 14 July 2000)*

**A failure in the compensate proliferation of pancreatic β-cells, as the primary pathogenic event, has been reported in OLETF rat, a model of NIDDM. The aim of the present study is to define whether the β-cell defect is attributed to the fetal stage islet development, if so, whether the defect involves down regulation of PDX-1 protein expression. Morphological changes, β-cell function, and the expression of PDX-1 protein were examined in the cultured fetal islet-like cell clusters (ICCs) from OLETF rats along with their diabetes-resistant control counterpart LETO rats in the presence of 5.5 or 11.1 mM glucose for 48, 72, 96, and 120-hr, respectively. We have observed four abnormalities in the ICCs of OLETF rats. First, a defective morphogenesis was noted during the 72 to 120-hr ICC culture, a period characterized by a dramatic increase in both β-cell and non-β-cell (α, δ, and PP) populations in control rats. This defective morphogenesis was demonstrated by a growth retardation of epithelial stratification and poor development of both β-cell and non-β-cell masses along with a parallel decline in relevant islet hormone contents. Second, a functional defect was characterized by failure to response to glucose during the 96 to 120-hr-cultured ICCs. Third, the ultrastructural analysis revealed a significant reduction in the number of secretory granules. Four, Western blot analysis showed a significant decrease of PDX-1 protein expression in the OLETF ICCs cultured in 11.1 mM glucose for 48 to 72-hr and in 5.5 mM glucose for 120-hr. Therefore, we concluded that during the fetal stage of islet development, OLETF rats exhibit both morphological and functional defects.**

*Keywords*: OLETF rat; NIDDM; PDX-1; GSIS; Fetal ICCs; Morphogenesis

*Abbreviations*: ICCs, Islet-like cell clusters; E17, 17 day embryo; PBS, phosphate-buffered saline; IRI, radioimmunoassay of insulin immunoreactivity; IRG, glucagon immunoreactivity; GSIS, glucose-stimulated insulin secretion; KRBB, Krebs-Ringer bicarbonate buffer

### INTRODUCTION

Evidence has shown that insulin resistance is a prerequisite for states of glucose intolerance to

*Address for correspondence: Research Institute for Children, 520 Elmwood Park Blvd. Suite 160, Harahan, LA 70123. Tel.: 504-734-1640, Fax: 504-733-4036, e-mail: mlan@childhealth-research.org develop significant fasting hyperglycemia, but clinical diabetes does not develop until pancreatic $\beta$-cells are no longer able to compensate or insufficiently compensate for the abnormal metabolic demand for insulin. [1–4] This suggests that diabetes mellitus represent a failure in the adaptation of pancreatic $\beta$-cell renewal and growth. Furthermore, morphorgenic studies have shown that pancreatic $\beta$-cells were originated from the growth and differentiation of pancreatic stem cells of duct. During the period of E17 to birth, an accelerated growth of islet cell populations is evident by both an elevated level of cell proliferation and the presence of large aggregated ductal epithelial cells, which subsequently differentiated into specific islet cell populations. [5–7] These processes are closely correlated with the transcriptional regulation of cell-specific gene expression. [8]

Otsuka Long-Evans Tokushima Fatty (OLETF) rats develop glucose intolerance with hyperinsulinemia at adulthood. [9] In earlier studies, we have shown that insulin resistance was apparent at 12 weeks of age, [10] prior to the impairment of pancreatic $\beta$-cell function. [11] Furthermore, the pancreatic $\beta$-cells of OLETF rats failed to compensate for a higher rate of $\beta$-cell proliferation by a partially pancreatectomy procedure as compared to the control rats. [12] The results implied that the pancreatic $\beta$-cells of OLETF rats were unable to compensate for changes in functional demand, once diabetogenic factors were sustained. However, it is unclear whether the islet cell defects in the OLETF rats are originated in a failure at the fetal stage. If so, whether a homeodomain-containing transcription factor, PDX-1, is associated with the defect in islet cell development since PDX-1 plays an essential role in the regulation of morphogenesis of pancreatic epithelium and the progression of endocrine cell differentiation. [13–16] To test this hypothesis, we investigate the differences in morphogenesis, functional role, and the PDX-1 expression level in the cultured fetal islet-like cell clusters (ICCs) from OLETF rats and their diabetes-resistant counterparts, Long-Evans Tokushima Otsuka (LETO) rats. [17]

### MATERIALS AND METHODS

### Animals

The OLETF rats and their control counterpart LETO rats (four weeks of age) were obtained from the Tokushima Research Institute, Otsuka Pharmaceutical Company. The animals were maintained in our animal facility under specific pathogen-free condition (Institute of Animal Experimentation, Tokushima University) until the age of 12 weeks for mating. All the studies were conducted with primiparous pregnancy rat. Pregnancy was timed from day 0 beginning in the morning when vaginal smears were monitored for the first presence of sperm. The non-fasting blood glucose and insulin levels were measured weekly at 4–5 p.m. after pregnancy. The animals were fed with standard rat chow (Oriental Yeast, Tokyo, Japan) and tap water *ad libitum*. The temperature ($21 \pm 2$°C), humidity ($55 \pm 5$°C), and lighting (07:00–19:00) were strictly controlled.

### Preparation and Culture of Fetal Islet-like Cell Clusters (ICCs)

On day 21 of gestation, pregnant rats were sacrificed by cervical dislocation after anesthetized with ether and the fetuses were rapidly removed. The fetal pancreases were finely minced and transferred to glass vials containing 4 ml of Hank's balanced salt solution with 2 mg/ml collagenase (S-1, Nitta Gelatin, Tokyo, Japan). The tissue fragments were shaken rapidly at 37°C and digested for approximately 2 min. The digest was then plated on a 60 mm petri dish (Steriline, Teddington, UK) to allow cells to attach to the plate. The cells were cultured at 37°C in a humidified atmosphere of 5% $CO_2$ in ambient air in Dulbecco's Modified Eagle

Medium supplemented with 10% fetal bovine serum (Laboratories, Logan, UT), 100 U/ml benzylpenicillin and 0.1 mg/ml streptomycin for the first 24 hr. At this time the culture medium was changed to RPMI-1640 medium containing the same supplements as above. Various concentrations of glucose were added at the beginning of culture and maintained until the end of each experimental time point. Over a period of 48-hr, three-dimensional cell aggregates were formed and referred to as ICCs. The ICCs consist predominantly of epithelial cells and a limited number of cells expressing islet hormones. The ICCs were harvested at 48-hr and cultured with fresh medium daily for 72, 96, and 120-hr, respectively.

### Hormone Content

A group of 10 ICCs were pooled, washed twice with PBS, resuspended in 200 µl of distilled water, and homogenized by sonication. The DNA content of a 50 µl aliquot of the homogenate was measured by fluorophotometry [18] using a calf thymus DNA (Sigma) as standard. The remainder of the homogenate was mixed with 300 µl of cold-acidified ethanol (0.18 M HCl in 95% ethanol) for an overnight extraction at 4°C. After centrifugation at 3000 rpm for 30 min, the supernatant was dried down and stored at −20°C for radioimmunoassay of insulin immunoreactivity (RIA) and glucagon immunoreactivity (IRG).

### Glucose-stimulated Insulin Secretion (GSIS)

Insulin secretion was measured for a group of 10 pooled ICCs in a transwell (New Transwell™ - Clear, Costar Scientific Corporation Cambridge, MA). The ICCs were preincubated in 1 ml of Krebs-Ringer bicarbonate buffer (KRBB) containing 143.5 mM $Na^+$, 5.8 mM $K^+$, 2.5 mM $Ca^{2+}$, and 25 mM $CO_3^{2-}$, 0.3% bovine serum albumin (BSA, Fraction V, ICN, Lisle, IL) and 3.3 mM glucose at 37°C, in an atmosphere of 95% $O_2$ : 5% $CO_2$ for 30 min. After preincubation, transwells containing ICCs were incubated for 2 hr in 1 ml KRBB. During the 1st hour, the medium was supplemented with 3.3 mM glucose. Subsequently, the transwell was transferred to a medium containing 27.7 mM glucose for the 2nd hour incubation. At the end of the incubations, aliquots were removed and stored at −20°C for IRI assay. The remaining ICCs were subjected to DNA content analyses using the same procedure as described above. In all cases, duplicates were performed for each experimental condition.

### Analytical Methods

Blood glucose values were determined by a glucose oxidase method (Toecho Super, Kyoto Daiichi Kagaku, Kyoto, Japan). IRI was determined using a commercially available IRI kit (Eiken Kagaku Co., Tokyo, Japan) with rat insulin (Novo Nordisk A/S, Bagsvaerd, Denmark) as a standard. IRG was measured using antiserum OAL-123 (Otsuka Pharmaceutical, Tokushima, Japan) that recognizes the free C-terminal end of glucagon but not glicentin or oxyntomodulin. [19]

### Western Blot Analysis

ICCs were prepared as described above and washed twice with PBS. The ICC pellet was homogenized by sonication in lysis buffer. After centrifugation at 15,000 rpm for 15 min, the supernatant was frozen. The protein concentration was determined using a micro BCA protein assay kit (PIERCE, Rockford, IL) with albumin as a standard (PIERCE, Rockford, IL). Ten microgram of proteins were separated by a 10% SDS-PAGE and transferred to a nitrocellulose membrane. The nitrocellulose membrane was blocked with 3% BSA in PBS for 90 mins and incubated at 4°C overnight in TBS buffer (20 mM Tris-HCl, 150 mM NaCl, pH 7.4)

containing a 1:5000 dilution of anti-PDX-1 antiserum. The PDX-1 antibody was generated by immunizing a rabbit with a synthetic peptide $_{269}$SPQPSSIAPLRPQE$_{282}$.[20] The blot was washed three times in TBS with 0.1% Tween-20 (TBS-T). The membrane was then incubated for 60 min at room temperature in TBS containing a 1:5000 dilution of a donkey anti-rabbit IgG horseradish peroxidase antibody (Amersham, International plc, Buckinghamshire, England). After three 30 min washes with TBS-T, immunoreactive bands were visualized by incubation with luminol (ECL® Western blotting Kit; Amersham, Tokyo, Japan). Quantification was achieved by densitometric scanning.

### Immunostaining

The ICCs were fixed in 10% buffered formalin for 25 min and then embedded in paraffin following a standard protocol. Two sets of three serial sections (3–5 μm thick) were obtained at an interval of 250 μm. Sections were immunostained using commercial ABC kits (Vector Laboratories Inc. Burlingame, CA). The primary antibodies used were polyclonal guinea pig anti-porcine insulin antibody (1:400, Dako Carpinteria, CA) and a mixture of antibodies against glucagon (rabbit anti-porcine glucagon, 1:1000, Dako Carpinteria, CA), somatostatin (rabbit anti-synthetic somatostatin, 1:2000, Monosan Synbio, The Netherlands) and pancreatic polypeptide (rabbit anti-bovine pancreas polypeptide, 1:4000, Monosan Synbio, The Netherlands). Afterwards, the sections were visualized by either 3,3′-diaminobenzidine or alkaline phosphatase substrate (Vector Red™, Vector Laboratories Inc. Burlingame, CA). Methyl green staining was used to reveal the duct-like structure or epithelial stratification. The profile area of either $\beta$-cell or non-$\beta$-cell mass, as a percentage of the total ICCs profile area, was quantified at an original magnification of 400$x$ on a monitor screen using an Olympus microscope connected to a color video camera and an image analysis system. The images were calibrated using images of a stage micrometer taken at the appropriate magnifications. A total of 30–40 ICCs (profile area > 2500 $\mu m^2$) were measured for each time period in the experiments.

### Electron Microscopy

For electron microscopy, the ICCs were fixed in 2.5% (vol/vol) glutaraldehyde dissolved in PBS (pH 7.2) for 2 hr at 4°C, and postfixed with 1% $OsO_4$. After dehydration, specimens were embedded in Epon 812. Ultrathin sections were prepared by mounting on one-hole grids and stained with uranyl acetate followed by lead citrate. Examination was preformed on a TEM HITACHI 7000 at an accelerating voltage of 60 kV.

### Statistical Analyses

Results were expressed as means ± SEM. For relative $\beta$-cell mass (%) and non-$\beta$-cell mass (%), a 10% adjusted means ± SEM was presented since the distribution of variables was skewed. Statistical significance was determined using either the Student's $t$-test (paired or unpaired) or analysis of variance (ANOVA). The level of significance was set at $P < 0.05$. All data were analyzed with a Macintosh SPSS® software package, version 6.0 (SPSS Inc., Chicago, IL).

### RESULTS

### *In Vivo* Metabolic State of OLETF and LETO Rats During Pregnancy

In order to ascertain that the fetal pancreas development was not affected by maternal hyperglycemia during pregnancy, we have measured the fasting blood glucose and plasma insulin level during the period of pregnancy (Tab. I). No significant difference of blood

TABLE I Non-fasting blood glucose and plasma insulin of OLETF and LETO rats during the pregnancy

| Pregnancy | Glucose (mM) | Insulin (pM) |
|---|---|---|
| | OLETF ($n = 10$) | |
| 1wk | 7.7 ± 0.2 | 350.7 ± 53.3 |
| 2wk | 5.4 ± 0.2 | 310.2 ± 30.5 |
| 3wk | 5.8 ± 0.4 | 298.1 ± 47.4 |
| | LETO ($n = 10$) | |
| 1wk | 7.3 ± 0.1 | 349.7 ± 60.7 |
| 2wk | 5.9 ± 0.3 | 301.1 ± 20.5 |
| 3wk | 4.7 ± 0.3 | 299.1 ± 57.4 |

Data presented as means ± SEM. No significant difference in non-fasting blood glucose and plasma insulin of OLETF and LETO rats were found during the pregnancy.

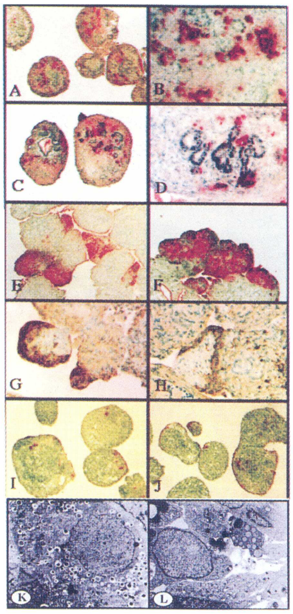

FIGURE 1 Morphological development of fetal ICCs cultured in the presence of 11.1 mM glucose. Immunostaining was performed by ABC method. $\beta$-cells were stained in red and non-$\beta$-cells ($\alpha$, $\delta$, and PP) were stained in brown. Methyl green staining shows the epithelial structure. A to H represent the ICCs from LETO rats and I to J show the ICCs from OLETF rats. Different culture times were shown as, 72h-cultured ICCs: A (100X), and B (400X); 96h-cultured ICCs: C (100X) and D (400X); 120h-cultured ICCs: E (100X), F (400X), G (400X), H (400X), I (100X), and J(100X). Electron photomicrographs are shown in K (5000X, LETO ICCs cultured for 120-hrs) and L (5000X, OLETF ICCs cultured for 120-hrs). (See Color Plate I).

glucose and plasma insulin level was found between the OLETF and LETO rats suggesting that fetal pancreas development was under normoglycemic condition.

### Morphological Development of Fetal ICCs

The fetal ICCs cultured in a medium containing 5.5 mM glucose showed no dramatic differences in the number, size, and shape between the OLETF and LETO during 48 to 96-hr culture. However, when the glucose concentration in the medium was increased to 11.1 mM, an outgrowth of islet was observed at 72-hr of the ICCs from LETO rats and the number of cell clusters ($< 100\,\mu m$ in diameter) increased with culture time. In contrast, the ICCs from OLETF rats remain rounded, enlarged and regular shaped.

As shown in Figure 1, three distinct patterns of morphological development were observed in the LETO ICCs cultured in 11.1 mM glucose. First, for the 48- to 72-hr culture (1-A and 1-B), large aggregated epithelial cells were evident in some cell clusters and positive islet hormone staining was observed for either a single cells or for a small groups of positive cells, which were scattered within the epithelium. The hormone-expressing cells were found to gather as small cell groups that appeared to be budding out from the ICCs without any well-defined islet cell architecture. However, the epithelial structure showed marked variations between different ICCs. Second, for the 72- to 96-hr culture, some of the ICCs showed epithelial stratification with a portion of the cells arranged in a duct-like structure (1-C). Immunostaining for islet hormones demonstrated insulin-positive (1-D) and non-$\beta$-cell hormone-positive cells (not shown) scattered in duct-like structured cells. In addition, the hormone-positive cell outgrowths were more commonly encountered in the peripheral portion of the ICCs. Third, for the 96-to 120-hr culture (1-E and 1-F), typical islets were formed, budding out from the ICCs, consisting of $\beta$-cells and the peripheral distribution of non-$\beta$-cells (1-G and 1-H). Since the aggregated epithelial cells, duct-like structure, and typical islets budding out from the ICCs appear in some of the clusters at the same culture time, it is unclear whether these three patterns represent three consecutive steps in the development of ICCs *in vitro*. Based upon our observation, it is clear that these three patterns of morphological development are influenced by the glucose concentration in culture medium. The morphological development of the ICCs cultured in 5.5 mM glucose, such as $\beta$-cell mass, epithelium growth, and islet budding, appears to be slower than those of the ICCs cultured in 11.1 mM glucose (data not shown). In contrast to the LETO ICCs, three dramatic differences were evident in ICCs from the OLETF rats (1-I and 1-J), including retarded epithelium growth, a lower number of islet hormone-positive cells, and rarely observed islet budding out from the ICCs. Upon further examination of ultrastructure of ICCs using electron microscope, a striking reduction in the number of secretory granules was found in the OLETF ICCs (1-L), in contrast to the LETO counterpart (1-K).

We also examined the hormone contents and islet cell masses in ICCs. Figure 2 shows the changes in insulin and glucagon contents in ICC extracts as the absolute values relative to the corresponding DNA content. For 5.5 mM glucose culture, the insulin content in the LETO ICCs appeared to be higher than those of the OLETF ICCs (2-A). Similarly, the glucagon content in the LETO ICCs was higher at each experimental point than those of the OLETF counterparts. However, the extent was not significant because of the large individual variations (2-C). In contrast, the 11.1 mM glucose culture has a significant increase in insulin content (2-B) in the LETO ICCs from 72- to 120-hr (a 2.1-fold increase for 72-hr, a 3.3-fold increase for both 96-hr and 120-hr, as compared with the OLETF counterparts). Also, the glucagon content (2-D) in the LETO ICCs showed a 3.1-fold increase for 72-hr, a 4.0-fold increase

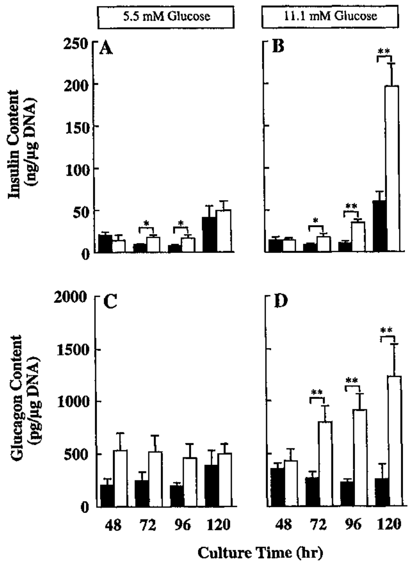

FIGURE 2 Insulin and glucagon contents of ICCs. ICCs from OLETF (■) and LETO (□) rats were measured for their insulin and glucagon contents and normalized with their DNA content. ICCs were cultured either in the presence of 5.5 mM glucose (A and C) or 11.1 mM glucose (B and D) for 48, 72, 96 and 120-hr, respectively. Data are presented as mean ± SEM from 6 experiments. $^{*}P < 0.05$, $^{**}P < 0.01$.

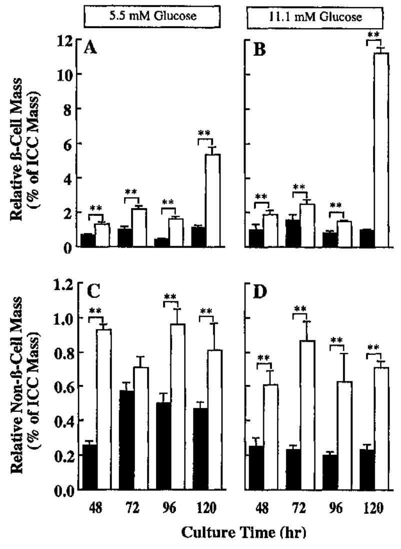

FIGURE 3 $\beta$-cell mass and non-$\beta$-cell mass of ICCs. ICCs from OLETF (■) and LETO (□) rats cultured either in 5.5 mM glucose (A and C) or 11.1 mM glucose (B and D) for 48, 72, 96 and 120-hr, respectively, were calculated for $\beta$-cell mass and non-$\beta$-cell mass as described in Materials and Methods. Values are expressed as the 10% adjusted mean percentage of $\beta$-cell mass, or non-$\beta$-cell mass profile area relative to the ICCs mass profile area. The bars represent SEM from 30–40 ICCs. $^{**}P < 0.01$.

for 96-hr, and a 4.8-fold increase for 120-hr, as compared with the OLETF counterparts. The insulin and glucagon content data is consistent with both $\beta$-cell mass (3-A and 3-B) and non-$\beta$-cell mass (3-C and 3-D) from the LETO ICCs, as a percentage of the total ICC mass. They are significantly higher than those from the OLETF counterparts. Furthermore, ICCs from the LETO rats, cultured in 11.1 mM glucose produced a higher $\beta$-cell mass than those of the culture maintained in 5.5 mM glucose.

### Functional Maturation of Cultured ICCs

We monitored the functional maturation of cultured ICCs in response to glucose stimulation in both OLETF and LETO rats (Fig. 4). It is apparent that higher glucose culture (11.1 mM) facilitates the maturation process of fetal ICCs suggesting that glucose is a potent stimulant for islet cell growth and differentiation. In LETO rats, a significant insulin response was observed in both 96 and 120-hr cultured ICCs. However, the intensity of GSIS is much higher in the ICCs cultured in 11.1 mM glucose than in the 5.5 mM glucose culture. In contrast, GSIS in OLETF ICCs failed to response except the ICCs cultured in 11.1 mM glucose for 120-hr. Furthermore, the level of insulin response in OLETF ICCs was much lower than the LETO ICCs. The GSIS indicated that glucose is a potent stimulant to facilitate fetal islet cell maturation and the OLETF ICCs failed to mature into functional islets.

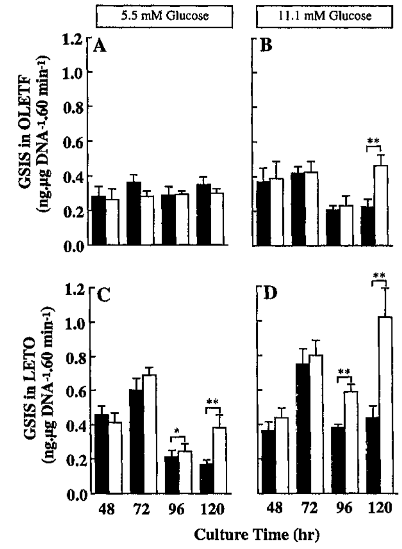

FIGURE 4 Glucose-stimulated insulin secretion. The ICCs from OLETF (A and B) and LETO (C and D) were cultured either in the presence of 5.5 mM glucose or 11.1 mM glucose for 48, 72, 96 and 120-hr, respectively. Insulin secretion in response to glucose stimulation for 2 hr consecutive incubations in KRB buffer with 3.3 mM glucose (■) for the first hour and 27.7 mM glucose (□) for the second hour. Insulin secretion was normalized with DNA content. Data are presented as mean ± SEM from 6 experiments. $^{*}P < 0.05$, $^{**}P < 0.01$.

### Expression of PDX-1 Protein in Cultured ICCs

Comparing the expression level of the PDX-1 protein between OLETF ICCs and LETO ICCs were shown in two culture conditions with 5.5 and 11.1 mM glucose concentrations (Fig. 5). PDX-1 was identified as a 46 kDa protein in ICCs by Western blotting with PDX-1 antiserum.[19] In 11.1 mM glucose culture (Fig. 5B), PDX-1 expression is significantly higher in normal LETO ICCs than in OLETF ICCs. Particularly, PDX-1 levels are higher at 48 and 72-hr culture, which probably attribute to the expression of PDX-1 in islet precursor cells. In a lower glucose

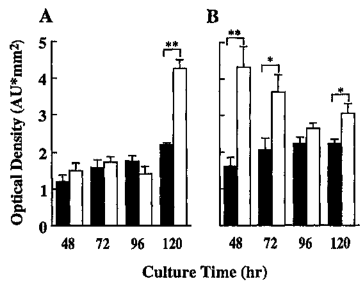

FIGURE 5 PDX-1 protein expression in ICCs. Western blot analysis of PDX-1 protein in the ICCs from OLETF (■) and LETO (□) rats. The ICCs were cultured either in the presence of 5.5 mM glucose (A) or 11.1 mM glucose (B) for 48, 72, 96 and 120-hr, respectively. Quantification of PDX-1 protein expression is shown by densitometer from 5 experiments. $^{*}P < 0.05$, $^{**}P < 0.01$.

concentration (5.5 mM) (Fig. 5A), the maturation of ICCs seems to be slower than 11.1 mM glucose. The expression level of PDX-1 only increased after 120-hr culture in LETO ICCs, which correlated well with the $\beta$-cell mass expansion. The OLETF ICCs expressed low levels of PDX-1 throughout the 5.5 mM glucose culture.

### DISCUSSION

The results presented here revealed defects in the development of ICCs from the OLETF rats, particularly in response to a high glucose culture. This defect was demonstrated as a growth retardation of the epithelial stratification and poor development in both $\beta$-cell and non-$\beta$-cell masses along with a parallel decline in relevant islet hormone contents. The similar abnormality in the fetal islets is generally observed only in manifest diabetic mothers with considerable hyperglycemia.[21,22] However, the cause of this defect is still unclear since we were unable to detect any abnormalities in the non-fasting blood glucose and plasma insulin level in the OLETF rats during pregnancy. The incidence of diabetes in the OLETF model is not homogenous and the incidence of diabetes between the male and female rats are 86% and 0% at the age over 23 weeks old,[9] but the female OLETF rats were shown to be potentially diabetic.[23,24] The sexual dimorphism in the incidence of diabetes mellitus in the OLETF model may attribute to the ovarian hormone induced $\beta$-cell hypertrophy in female rats.[25] A characteristic feature of male pancreas from diabetic OLETF rat exhibits fibrotic connective tissue proliferation.[11] In this study, we focus on fetal model of OLETF rat pancreas development. Therefore, the fetal ICCs from both sexes were used in this study.

The morphological observations in the control rats, namely, the development of epithelial stratification and duct-like structure, as well as subsequent insulin-positive cell outgrowths from the undifferentiated epithelial cell clusters are consistent with islet neogenesis.[5–7] The development of pancreatic islets appears to be regulated by a variety of growth factors, whereas glucose is a cardinal secretory and mitogenic stimulus for pancreatic $\beta$-cells both *in vitro* and *in vivo*.[26] In control rats, 11.1 mM glucose produced higher $\beta$-cell mass which was accompanied by an increase in insulin content and GSIS than observed for a culture in 5.5 mM glucose. It is conceivable that ICCs response to high glucose concentration to undergo islet cell differentiation *in vitro*.[27] Furthermore, the genetic background is of considerable importance in determining the proliferative response of $\beta$-cells to a change in environmental factor.[28] Therefore, we speculated that a limited potential to undergo islet cell differentiation could be attribute to a failure involved in the fetal model of the OLETF rat, which might be genetically predisposed.

It has been reported that rat fetal islets, up to 21-days, show an immature response to glucose-stimulation[29,30] and these immature islets need to undergo additional development during the postnatal period to become mature islet cells.[31] Our data on the GSIS showed a progressive increase ratio in response to glucose stimulation as coupled with the development of $\beta$-cell mass in the ICCs from control LETO rats instead of OLETF rats. Similarly, the changes in basal insulin secretion and content of insulin were also consistent with the changes in the proportion of $\beta$-cell mass within ICCs. It has been reported that 11.1 mM glucose resulted in significant increase in the volume of $\beta$-cell mitochondria, secretory granules, and endoplasmic reticulum, which contribute to an increased in insulin content and further increase in insulin secretion rate.[29] A delayed maturation of the fetal $\beta$-cells was also reported due to a lack of glucose-stimulated $\beta$-cell mass development with low rate of $\beta$-cell division observed in the offspring of manifest diabetic rat mothers.[21] Both of the studies further support that OLETF rats exhibit a defect involving functional maturation coupled with morphological development. Taken together, these findings suggest that poor development of functional $\beta$-cell mass may be a result of delayed maturation of fetal $\beta$-cells in the OLETF model rat.

PDX-1 has been shown to be essential for pancreatic development. PDX-1-deficient mice failed to develop a pancreas.[13,15] During the early development, PDX-1 was detected in most pancreatic epithelial cells, and later only the islet hormone-positive cells and a few ductal cells were found to be positive for PDX-1[32,33] suggesting that PDX-1 is an important factor in pancreatic development and in mature $\beta$-cells. In the present study, we revealed that the PDX-1 expression in the ICCs from OLETF rats was significantly lower than that of the control rats. Although we have little direct evidence to demonstrate PDX-1 expression in islet precursor cells in this report, it is reasonable to speculate that a failure of PDX-1 expression may hinder the islet precursor cells from fully proliferating and/or differentiating into mature endocrine cells. In the presence of 11.1 mM glucose, a dramatic increase of PDX-1 in the ICCs from the control rats in early culture period (48 to 72-hr) was characterized by the occurrence of large aggregated epithelial cells and the formation of duct-like structures. This suggests that the expression of PDX-1 may be required for the early inductive event leading to the formation of the pancreatic precursor cells and the subsequent endocrine cell differentiation and development *in vitro*. Another possible scenario will be that the main defect of OLETF rats is due to the inability of the mutant $\beta$-cells to proliferate. This decrease in $\beta$-cell proliferation results in a lower number of $\beta$-cells and, consequently, to a decrease in expression of PDX-1 per ICC. In order words, the ICCs have low PDX-1 because they have fewer $\beta$-cells. Therefore, it would be interesting to further analyze this defect during the embryonic development *in vivo*.

*Acknowledgements*

The authors thank Dr. Y. Kajimoto (Osaka, Japan) for kindly providing PDX-1 antiserum. This work was supported by grant (09671001 to K.S) and a grant-in-aid (P97152 to M.Z.) from the Ministry of Education, Science and Culture, Japan. The animals were supplied by Otsuka Pharmaceuticals (Tokushima, Japan). M.Z. was a recipient of a postdoctoral fellowship from the Japan Society for the Promotion of Science.

*References*

[1] Polonsky, K. S., Sturis, J. and Bell, G. I. (1996). Non-insulin dependent diabetes mellitus: a genetically programmed failure of the beta-cell to compensate for insulin resistance. *N. Engl. J. Med.*, **334**, 777–783.

[2] Kloppel, G., Lohr, M., Hablich, K., Oberholzer, M. and Heitz, P. U. (1985). Islet pathology and pathogenesis of type 1 and type 2 diabetes mellitus revised. *Surv. Synth. Pathol. Res.*, **4**, 110–125.

[3] Sjoholm, A. (1996). Diabetes mellitus and impaired pancreatic beta-cell proliferation. *J. Internal. Med.*, **239**, 211–220.

[4] Movassat, J., Saulnier, C., Serradas, P. and Portha, B. (1997). Impaired development of pancreatic beta-cell mass is a primary event during the progression to diabetes in the GK rat. *Diabetologia*, **40**, 916–925.

[5] Bouwens, L., Wang, R. N., DeBlay, E., Pipeleers, D. G. and Kloppel, G. (1994). Cytokeratins as markers of ductal cell differentiation and islet neogenesis in the neonatal rat pancreas. *Diabetes*, **43**, 1279–1283.

[6] Bouwens, L., Lu, W. G. and DeKrijger, R. (1997). Prolifration and differentiation in the human fetal endocrine pancreas. *Diabetologia*, **40**, 398–404.

[7] Kaung, H. C. (1994). Growth dynamics of pancreatic islet cell populations during fetal and neonatal development of the rat. *Dev. Dynamics*, **200**, 163–175.

[8] Medsen, O. D., Jensen, J., Blume, N., Petersen, H. V., Lund, K., Karlsen, C., Andersen, F. G., Jensen, P. B., Larsson, L. I. and Suerup, P. (1996). Pancreatic development and maturation of the islet beta-cell. Studies of pluripotent islet cultures. *Eur. J. Biochem.*, **242**, 435–445.

[9] Kawano, K., Hirashima, T., Mori, S., Saitoh, Y., Kurosumi, M. and Notori, T. (1992). Spontaneous long-term hyperglycemic rat with diabetic complications: Otsuka Long-Evans Tokushima Fatty (OLETF) strain. *Diabetes*, **41**, 1422–1428.

[10] Sato, T., Asahi, Y., Toide, K. and Nakayama, N. (1995). Insulin resistance in skeletal muscle of the male Otsuka Long-Evans Tokushima Fatty rat, a new model of NIDDM. *Diabetologia*, **38**, 1033–1041.

[11] Ishida, K., Mizuno, A., Zhu, M., Sano, T. and Shima, K. (1995). Which is the primary etiological event in Otsuka Long-Evans Tokushima Fatty rat, a model of spontaneous non-insulin-dependent diabetes mellitus, insulin resistance, or impaired insullin secretion? *Metabolism*, **44**, 940–944.

[12] Zhu, M., Noma, Y., Mizuno, A., Sano, T. and Shima, K. (1996). Poor capacity for proliferation of pancreatic beta-cells in Otsuka Long-Evans Tokushima Fatty rat. A model of spontaneous NIDDM. *Diabetes*, **45**, 941–946.

[13] Jonsson, J., Carlsson, L., Edlund, T. and Edlund, H. (1994). Insulin promoter-factor 1 is required for pancreas development in mice. *Nature*, **371**, 606–609.

[14] Offield, M. F., Jetton, T. L., Labosky, P. A., Ray, M., Stein, R. W., Magnuson, M. A., Hogan, B. L. and Wright, C. V. (1996). PDX-1 is required for pancreatic outgrowth and differentiation of the rostral duodenum. *Development*, **122**, 983–995.

[15] Ahlgren, U., Jonsson, J. and Edlund, H. (1996). The morphogenesis of the pancreatic mesenchyme is uncoupled from that of the pancreatic epithelium in IPF-1/PDX-1-deficient mice. *Development*, **122**, 1409–1416.

[16] Otonkoski, T., Cirulli, V., Beattie, M., Mally, M. I., Soto, G., Rubin, J. S. and Hayek, A. (1996). A role for hepatocyte growth factor/scatter factor in fetal mesenchyme-induced pancreatic beta-cell growth. *Endocrinology*, **137**, 3131–3139.

[17] Zhu, M., Mizuno, A., Kuwajima, M., Murakami, T. and Shima, K. (1998). Expression of PDX-1, a homeodomain-containing transcription factor, and development of fetal islet-like cell clusters in OLETF rat, a model of NIDDM. *Diabetes*, **47**, Suppl. 1, A261.

[18] Hinegardner, R. T. (1971). An improved fluorometric assay for DNA. *Anal. Biochem.*, **39**, 197–201.

[19] Nishino, T., Kodaira, T., Shin, S., Imagawa, K., Shima, K., Kumahara, Y., Yanaihara, C. and Yanaihara, N. (1981). Glucagon radioimmunoassay with use of antiserum to glucagon C-terminal fragment. *Clin. Chem.*, **27**, 1690–1697.

[20] Kajimoto, Y., Watada, H., Matsuoka, T., Kaneto, H., Fujitani, Y., Miyazaki, J. and Yamasaki, Y. (1997). Suppression of transcription factor PDX-1/IPF-1/STF-1/IDX-1 causes no decrease in insulin mRNA in MIN6 cells. *J. Clin. Invest.*, **100**, 1846

[21] Swenne, I. and Eriksson, U. (1982). Diabetes in pregnancy: Islet cell proliferation in the fetal rat pancreas. *Diabetologia*, **23**, 525–528.

[22] Kervran, A., Guillaume, M. and Jost, A. (1978). The endocrine pancreas of the fetus from diabetic pregnant rat. *Diabetologia*, **15**, 387–393.

[23] Hirashima, T., Kawano, K., Mori, S., Matsumoto, K. and Natori, T. (1995). A diabetogenic gene (ODB-1) assigned to the X-chromosome in OLETF rats. *Diabetes Res. Clin. Pract.*, **27**, 91–96.

[24] Shi, K., Mizuno, A., Sano, T., Ishida, K. and Shima, K. (1994). Sexual difference in the incidence of diabetes mellitus in Otsuka-Long-Evans-Tokushima-Fatty rats: effects of castration and sex hormone replacement on its incidence. *Metabolism*, **43**, 1214–1220.

[25] Zhu, M., Mizuno, A., Kuwajima, M., Ogino, T., Murakami, T., Noma, Y., Sano, T. and Shima, K. (1998). Ovarian hormone-induced beta-cell hypertrophy contributes to the homeostatic control of beta-cell mass in OLETF female rat, a model of type II diabetes. *Diabetologia*, **41**, 799–805.

[26] Sjoholm, A. (1997). Glucose stimulates islet beta-cell mitogenesis through GTP-binding proteins and by protein kinase C-dependent mechanisms. *Diabetes*, **46**, 1141–1147.

[27] Hellerstrom, C. and Swenne, I. (1991). Functional maturation and proliferation of fetal pancreatic beta-cells. *Diabetes*, **40** (Suppl. 2), 89–93.

[28] Swenne, I. and Andersson, A. (1984). Effect of genetic background on the capacity for islet cell replication in mice. *Diabetologia*, **27**, 464–467.

[29] Dudek, R. W., Kawabe, T., Brinn, J. E., O'Brien, K., Poole, M. C. and Morgan, C. R. (1984). Glucose affects *in vitro* maturation of fetal rat islets. *Endocrinology*, **114**, 582–587.

[30] Dudek, R. W., Freinkel, N., Lewis, N. J., Hellerstrom, C. and Johnson, R. C. (1980). Morphologic study of cultured pancreatic fetal islets during maturation of the insulin stimulus-secretion mechanism. *Diabetes*, **29**, 15–21.

[31] Roberts, H. R., Pettinati, J. D. and Bucek, W. (1954). A comparative study of human, cow, sow, and rat milk using paper chromatography. *J. Dairy Sci.*, **37**, 538–547.

[32] Stoffers, D. A., Heller, R. S., Miller, C. P. and Habener, J. F. (1999). Developmental expression of the homeodomain protein IDX-1 in mice transgenic for an IDX-1 promoter/lacZ transcriptional reporter. *Endocrinology*, **140**, 5374–5381.

[33] Oster, A., Jensen, J., Serup, P., Galante, P., Madsen, O. D. and Larsson, L. I. (1998). Rat endocrine pancreatic development in relation to two homeobox gene products (Pdx-1 and Nkx6.1). *J. Histochem. Cytochem.*, **46**, 707–715.

